# Sociodemographic Influences on Lumbar Disc Degeneration Severity and the Diagnostic Potential of Disc‑CSF Signal Ratio: Insights from a South East Asia Population Study

**DOI:** 10.5334/jbsr.3801

**Published:** 2025-03-12

**Authors:** Tze Hui Soo, Subapriya Suppiah, Anas Tharek, Tatt Quan Tan, Siti Anisah Koya Asrab Jailani, Adam Adnan

**Affiliations:** 1 Department of Radiology, Faculty of Medicine and Health Sciences, Universiti Putra Malaysia, Malaysia; 2 School of Medicine, Faculty of Medicine and Health Sciences, Universiti Putra Malaysia, Malaysia

**Keywords:** lumbar disc degeneration, disc–CSF signal ratio, magnetic resonance imaging, low back pain, Pfirrmann grading system

## Abstract

*Objectives:* This study investigates the relationship between lumbar disc degeneration and sociodemographic factors in a Malaysian cohort, focusing on the diagnostic potential of the disc–cerebrospinal fluid signal ratio (DCFR). With lumbar disc degeneration being a significant cause of low back pain, there is a need for simple yet effective diagnostic measures that are adaptable to diverse imaging conditions.

*Materials and methods:* A retrospective analysis was conducted on 182 patients presenting with low back pain. Magnetic resonance imaging (MRI) was used to assess disc degeneration using the Pfirrmann grading system, while the DCFR was calculated to quantify the severity of disc degeneration. Sociodemographic factors such as age, gender, and race were analyzed for their correlation with degeneration severity and DCFR.

*Results:* The DCFR showed a strong negative correlation with Pfirrmann grades, with older patients and males exhibiting more severe degeneration. Sociodemographic factors significantly influenced degeneration patterns, particularly in the older age groups, with Malays showing a higher prevalence of moderate to severe degeneration.

*Conclusion:* The DCFR provides a consistent and practical quantitative assessment of lumbar disc degeneration. It correlates well with traditional qualitative grading systems and is effective across various age group, making it a valuable tool for clinical and diagnostic applications in diverse populations.

## Introduction

**Figure 1:**
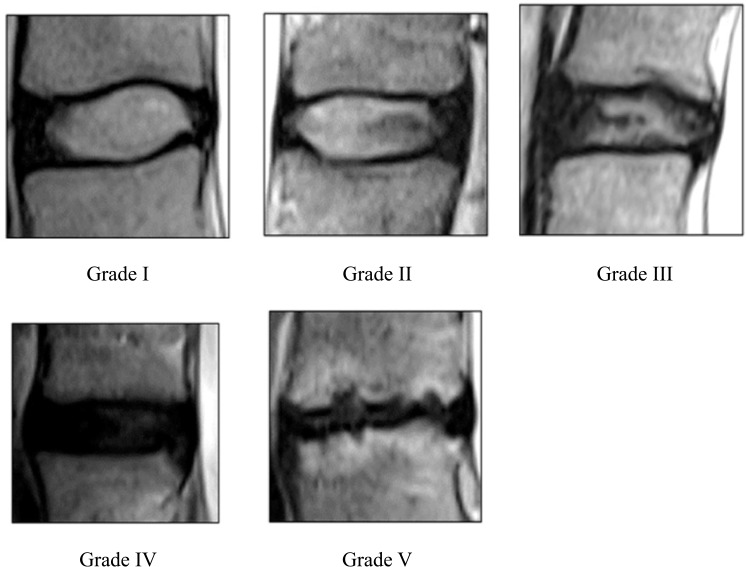
Reference images of lumbar spine intervertebral disc degeneration on based on Pfirrmann grading system (Adapted from Pfirrmann et al. (2001)).

Early degeneration of lumbar discs leads to chronic low back pain and is a significant health concern having substantial socioeconomic implications, notably due to increased morbidity, medical costs, and decreased productivity. In Malaysia, the prevalence of lumbar disc degeneration ranges from 10% to 63%, with a median of 37%, and higher rates have been observed in high‑risk groups, such as commercial vehicle drivers. This degeneration commonly occurs at the L3‑L4 and L5‑S1 vertebral levels [1]. The pathogenesis of disc degeneration involves radial fissures, annular tears, and a loss of water content in the nucleus pulposus and annulus fibrosus [2]. Lumbar disc degeneration often leads to secondary conditions such as disc herniation and degenerative lumbar instability [3]. The Pfirrmann grading system (Figure 1) is frequently used to assess the severity of disc degeneration based on T2‑weighted magnetic resonance imaging (MRI) images (T2WI), categorizing degeneration from grades I to V, with higher grades indicating more severe forms [4]. This grading system focuses on specific criteria such as MRI signal intensity (SI), disc structure, nucleus–annulus discrimination, and disc height. This may limit the comprehensive evaluation of disc degeneration, potentially overlooking important pathological changes such as endplate sclerosis, vertebral osteophytes, or disc herniation [5]. Although considered a reliable method for the assessment of disc degeneration, the Pfirrmann system often lacks sensitivity in detecting subtle and early‑stage changes that can occur insidiously. Moreover, the system is less reliable for the assessment of slow‑progressing degeneration, particularly in older populations.

Consequently, quantitative MRI techniques, such as T2 relaxation time and ADC value measurements, offer a more detailed evaluation of disc hydration and degeneration but can be resource intensive. In resource‑limited settings, measuring the disc–cerebrospinal fluid signal ratios (DCFR) has been proposed to be able to provide a more consistent and practical alternative assessments [6]. Thus, the aim of this study is to correlate the severity of lumbar disc degeneration based on Pfirrmann grades with the DCFR ratio in the Malaysian population. Additionally, it seeks to examine the influence of sociodemographic factors on the prevalence of lumbar disc degeneration, contributing to a broader understanding of spinal health.

## Materials and Methods

This retrospective cross‑sectional analysis utilized convenience sampling at our hospital. Relevant radiological images and sociodemographic data were collected from the hospital’s database between January and December 2022, following ethical approval from the institutional review board (JKEUPM‑2023‑209). The study included Malaysian adults aged 18–80 years, regardless of gender, presenting with low back pain of any etiology and duration (acute, subacute, and chronic). Exclusion criteria were incomplete MRI sequences, significant image artifacts, vacuum phenomenon, gross spinal deformities that included scoliosis, vertebra segmentation anomalies, congenital abnormalities, as well as annular tears, focal disc herniations, and traumatic disc injuries.

## Imaging Techniques

All the MRI examinations were conducted on a 3 Tesla Philips Ingenia scanner from the Netherlands, using a five‑channel lumbar coil and application of a standard lumbosacral spine protocol. Conventional MRI sequences, which included T1‑weighted imaging (T1WI) and T2‑weighted imaging (T2WI) in both sagittal and axial views from L1 to S3 vertebra levels were acquired. The T2WI sequence was obtained using a fast spin echo sequence with the following parameters: TR/TE = 3797/90 ms, slice thickness of 4 mm, gap of 0.4 mm, field of view (FOV) of 16 cm × 28 cm, matrix size of 400 × 254, with scan time of 5 minutes 11 seconds.

## MRI Image Analysis

**Figure 2:**
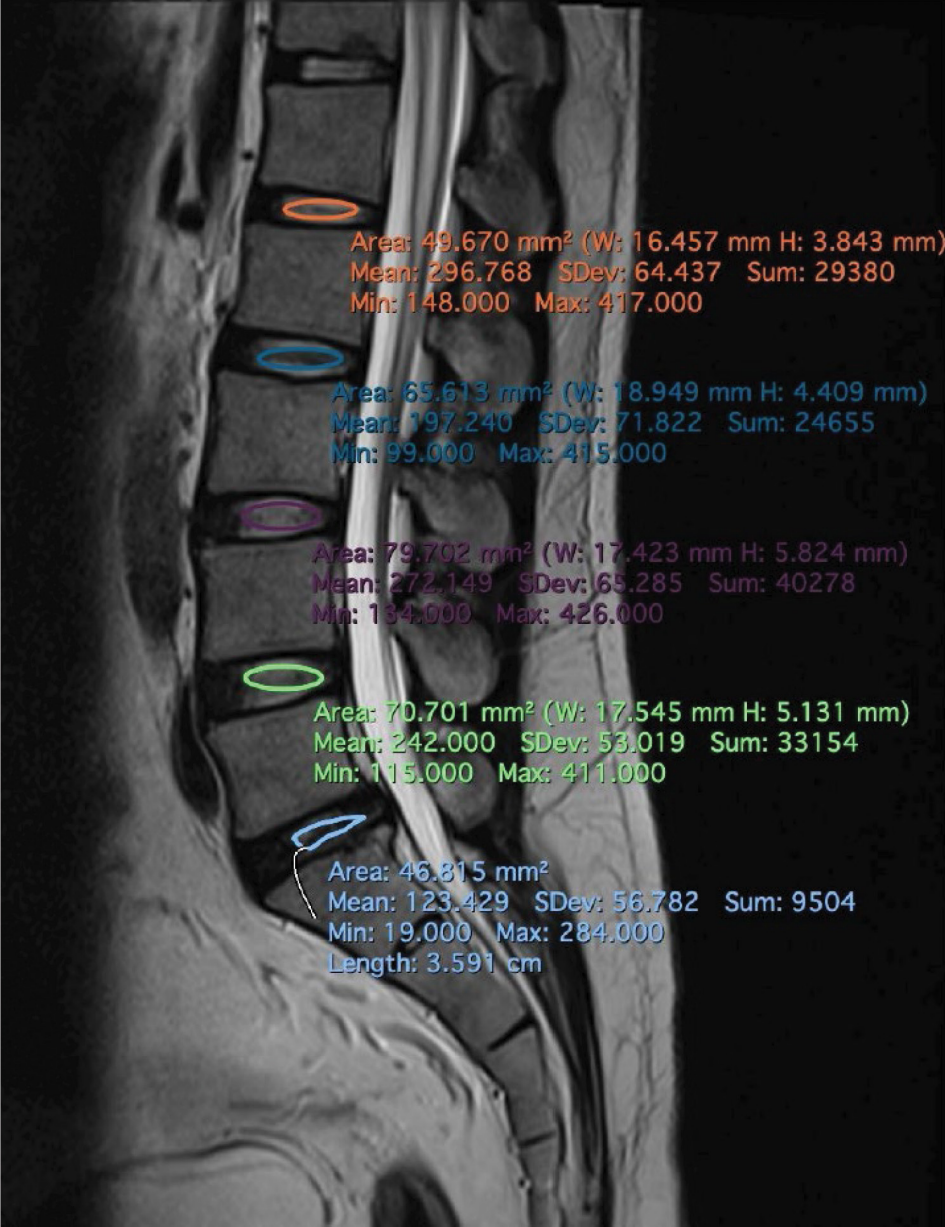
Schematic diagram of region of interest (ROI) from L1/L2 to L5/S1 intervertebral disc level. ROIs are selected as an oval area covered as much nucleus pulposus as possible.

The mid‑sagittal T2WI were independently reviewed by two radiologists, both with over 5 years of experience, respectively, using the Pfirrmann grading classification. Intervertebral discs from L1/L2 to L5/S1 were analyzed, and a consensus was reached by discussion for cases with differing grades. The reviewers were blinded to all the sociodemographic information. Mean signal intensity (SI) values for the lumbar intervertebral discs and cerebrospinal fluid (CSF) were measured on a DICOM image viewer. An oval region of interest (ROI) was manually drawn at each disc level, capturing as much of the nucleus pulposus as possible (Figure 2). In cases with grade V discs with collapsed heights, an irregular‑shaped ROI was used to encompass the remaining visible nucleus pulposus. The mean SI and standard deviation were calculated for each nucleus pulposus at the relevant lumbar levels. To reduce human error, two repeated ROIs were drawn at each disc level, and their average SI was computed. An additional ROI was placed on the brightest CSF region at the lumbar region to measure the CSF SI. Similarly, the CSF SI too was averaged from two repeated ROIs measurements. Finally, the intervertebral disc SI was divided by the CSF SI to calculate the disc–CSF signal ratio (DCFR = SI_disc_ / SI_CSF_).

## Statistical Analysis

Statistical analyses were performed using IBM SPSS version 29. The study’s independent variables were patients’ sociodemographic factors (age, gender, and race), while the dependent variables were the Pfirrmann grading system and DCFR. Descriptive analysis described patients’ characteristics and determined the prevalence of lumbar disc degeneration based on the Pfirrmann grading system. A Fisher’s Exact Test was used to assess the relationship between sociodemographic factors (gender and race) and the Pfirrmann grading system. One‑way analysis of variance (ANOVA) test was used to assess the relationship between sociodemographic factors (age) and the Pfirrmann grading system. To explore the relationship between sociodemographic factors and DCFR, Kruskal–Wallis and Mann–Whitney tests were utilized, respectively. The DCFR for each Pfirrmann grade was reported as the median with interquartile range (IQR) across all 910 discs. A Spearman rank correlation assessed the strength of the relationship between the Pfirrmann grading and DCFR and was reported as variance (*R*²). A *P* value < 0.05 was considered statistically significant for all analyses.

## Results

A total of 182 participants were recruited, and their sociodemographic profile is illustrated in Table 1. Females (63.2%) outnumber males (36.8%), indicating a higher prevalence of lower back pain among women. Racially, Malays dominated the sample at 84.1%, followed by Chinese (6.0%) and Indians (9.9%). Age wise, the highest representation was in the 60–69 years age group (29.1%), followed by 30–39 years (22.0%) and 50–59 years (18.1%). Younger adults (20–29 years) and those over 70 years were least represented at 5.5% and 11.5%, respectively.

**Table 1:** Sociodemographic analysis.

SOCIODEMOGRAPHIC CHARACTERISTICS		
	**Frequency (*n*)**	**Percentage (%)**
**Gender**		
Male	67	36.8
Female	115	63.2
**Race**		
Malay	153	84.1
Chinese	11	6.0
Indian	18	9.9
**Age group (years)**		
20–29	10	5.5
30–39	40	22.0
40–49	25	13.7
50–59	33	18.1
60–69	53	29.1
>70	21	11.5

**Figure 3:**
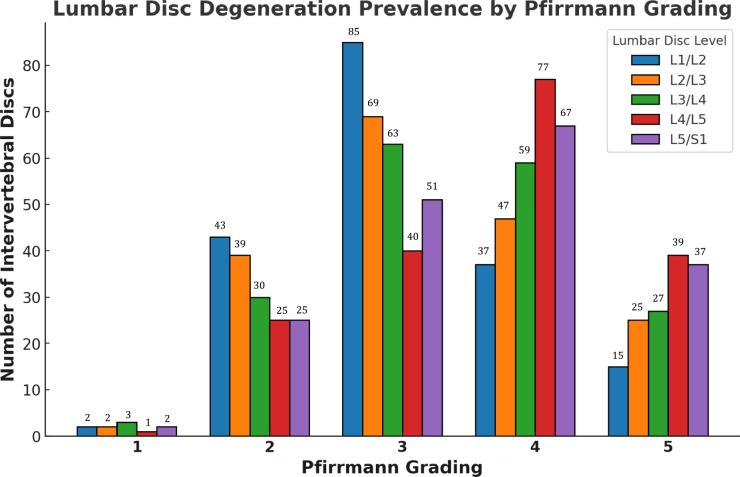
Lumber disc degeneration prevalence by Pfirrmann grading (based on the number of intervertebral disc).

Table 2 and Figure 3 provide an intricate overview of lumbar disc degeneration prevalence at our hospital, categorized by the Pfirrmann grading, across various lumbar spine levels (L1/L2 to L5/S1). Grade 3 degeneration predominated across all lumbar levels, indicating that moderate degeneration was the most common severity observed. Grade 4 degeneration was also significantly represented, particularly at the L4/L5 and L5/S1 levels, indicating that severe degeneration frequently occurred in the lower lumbar spine. Grade 2 degeneration was consistently present across all the lumbar spine levels. Grade 1 and grade 5 disc degeneration were detected least commonly.

**Table 2:** Lumbar disc degeneration prevalence by Pfirrmann grading (based on the number of intervertebral disc).

LUMBAR DISC LEVEL	PFIRRMANN GRADING	FREQUENCY (*N*)	PERCENTAGE (%)
L1/L2	1	2	1.1
	2	43	23.6
	3	85	46.7
	4	37	20.3
	5	15	8.2
L2/L3	1	2	1.1
	2	39	21.4
	3	69	37.9
	4	47	25.8
	5	25	13.7
L3/L4	1	3	1.6
	2	30	16.5
	3	63	34.6
	4	59	32.4
	5	27	14.8
L4/L5	1	1	0.5
	2	25	13.7
	3	40	22.0
	4	77	42.3
	5	39	21.4
L5/S1	1	2	1.1
	2	25	13.7
	3	51	28.0
	4	67	36.8
	5	37	20.3

**Figure 4:**
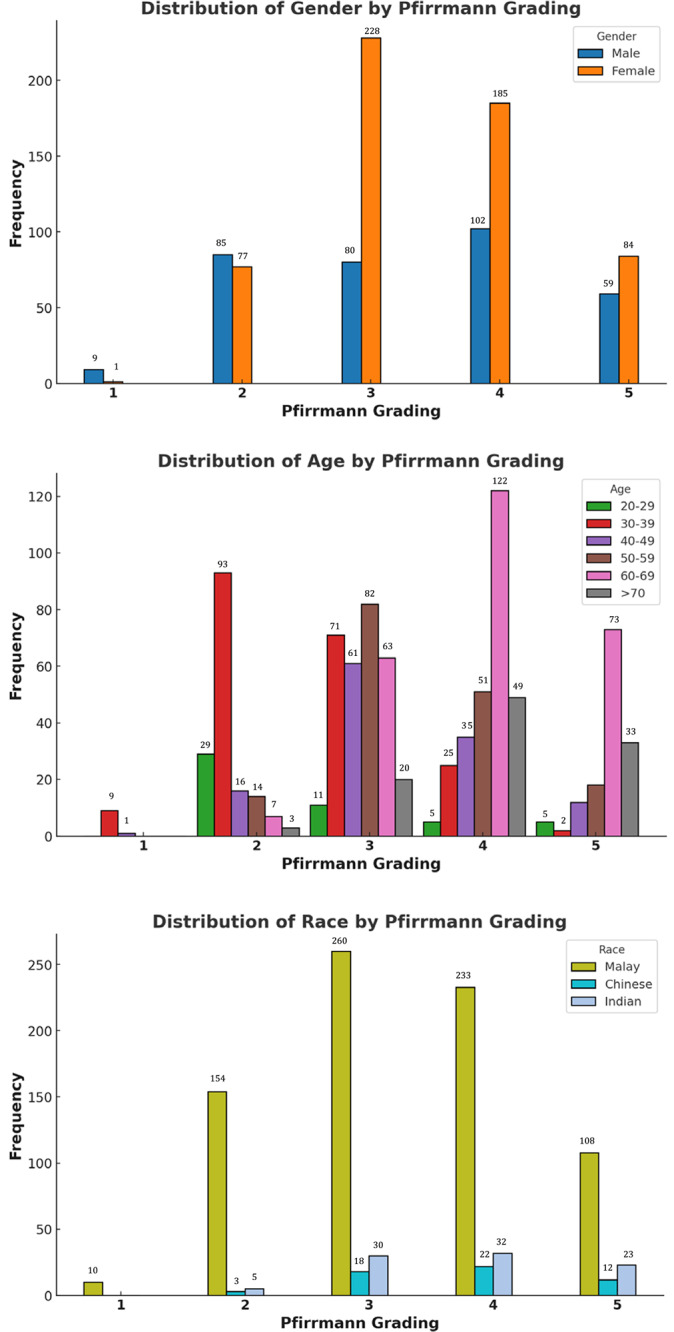
Relationship between sociodem ographic factors with Pfirrmann grading (based on the number of intervertebral disc).

The relationship between sociodemographic factors (age, gender, and race) with the Pfirrmann Grading is tabulated in Table 3 and Figure 4. The gender distribution of lumbar disc degeneration exhibited notable disparities (*P* value < 0.001). In males, there was a pronounced prevalence of severe degeneration, with 30.4% of cases classified as grade 4 and 17.6% as grade 5. The significant proportions in grades 2 (25.4%) and 3 (23.9%) imply a tendency among males to progress from mild to severe degeneration stages. Conversely, females predominantly exhibited moderate degeneration, with 39.7% of cases being categorized as grade 3 and 32.2% as grade 4. The incidence of severe degeneration (grade 5) was relatively lower, at 14.6%. Age‑specific trends demonstrated a clear and progressive correlation with advancing age (*P* value < 0.001). Individuals in the 20–29 years age group predominantly experience mild degeneration, with 58.0% classified as grade 2 with minimal representation at higher grades. As individuals transition into their 30s, moderate degeneration (grades 2 and 3) became more prevalent, indicating the initiation of progressive disc deterioration. The 40–49 years age group shows a marked increase in moderate‑to‑severe degeneration was observed, with 48.8% of cases being categorized as grade 3 and 28.0% as grade 4, respectively. This trend continued into the 50–59 years age group, where moderate degeneration remained dominant (grade 3 at 49.7%), alongside a significant rise in severe degeneration (grade 4 at 30.9%). In the 60–69 years and over 70‑year age groups, the prevalence of severe degeneration reached its peak, with > 70% having grade 4 and grade 5 disc degeneration.

**Table 3:** Relationship between sociodemographic factors with Pfirrmann grading (based on the number of intervertebral disc).

SOCIODEMOGRAPHIC FACTORS	PFIRRMANN GRADING					*P* VALUE
	1	2	3	4	5	
**Gender**						
Male	9 (2.7%)	85 (25.4%)	80 (23.9%)	102 (30.4%)	59 (17.6%)	< 0.001*
Female	1 (0.2%)	77 (13.4%)	228 (39.7%)	185 (32.2%)	84 (14.6)	
**Age (years)**						
20–29	0 (0.0%)	29 (58.0%)	11 (22.0%)	5 (10.0%)	5 (10.0%)	< 0.001*
30–39	9 (4.5%)	93 (46.5%)	71 (35.5%)	25 (12.5%)	2 (1.0%)	
40–49	1 (0.8%)	16 (12.8%)	61 (48.8%)	35 (28.0%)	12 (9.6%)	
50–59	0 (0.0%)	14 (8.5%)	82 (49.7%)	51 (30.9%)	18 (10.9%)	
60–69	0 (0.0%)	7 (2.6%)	63 (23.8%)	122 (46.0%)	73 (27.5%)	
> 70	0 (0.0%)	3 (2.9%)	20 (19.0%)	49 (46.7%)	33 (31.4%)	
**Race**						
Malay	10 (1.3%)	154 (20.1%)	260 (34.0%)	233 (30.5%)	108 (14.1%)	< 0.001*
Chinese	0 (0.0%)	3 (5.5%)	18 (32.7%)	22 (40.0%)	12 (21.8%)	
Indian	0 (0.0%)	5 (5.6%)	30 (33.3%)	32 (35.6%)	23 (25.6%)	

Racial differences revealed significant disparities among the Malay, Chinese, and Indian populations (*P* value < 0.001). The Malay group exhibited the highest prevalence in moderate degeneration (grade 3, 34.0%) and severe degeneration (grade 4, 30.5%), respectively indicating a wide spectrum of degeneration severity within this population. The Chinese population showed a higher proportion of severe degeneration (grade 4, 40.0%) alongside moderate degeneration (grade 3, 32.7%), suggesting a trend toward more advanced stages of degeneration. Similarly, the Indian population demonstrated a balanced distribution between moderate (grade 3, 33.3%) and severe degeneration (grade 4, 35.6%), indicating a pattern akin to the Chinese group.

**Figure 5:**
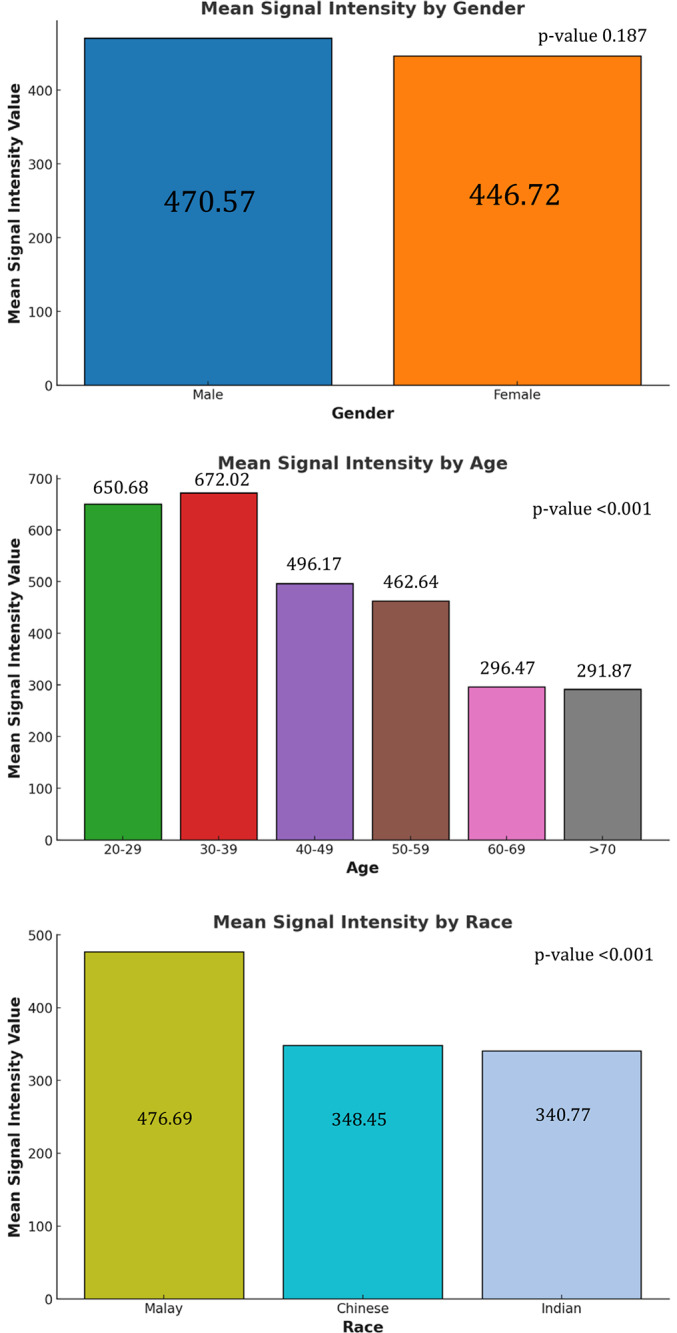
Relationship between sociodem ographic factors with mean signal intensity value.

The analysis of the mean SI value of lumbar disc (Figure 5) revealed that males have a mean rank of 470.57, while females had a mean rank of 446.72. Despite the slightly higher mean rank observed in males, this difference did not achieve statistical significance (*P* value = 0.187). The impact of age on the mean SI value was notably significant (*P* value < 0.001). The age group 30–39 years exhibited the highest mean rank of 672.02, followed closely by the 20–29 years group with a mean rank of 650.68. As age increased, a noticeable decline in mean ranks was observed. Racial differences in the mean SI value revealed statistically significant disparities (*P* value <0.001). Malay participants exhibited the highest mean rank of 476.69, indicating comparatively better disc health. In contrast, Chinese and Indian participants showed mean ranks of 348.45 and 340.77, respectively, reflecting more severe disc degeneration.

**Figure 6:**
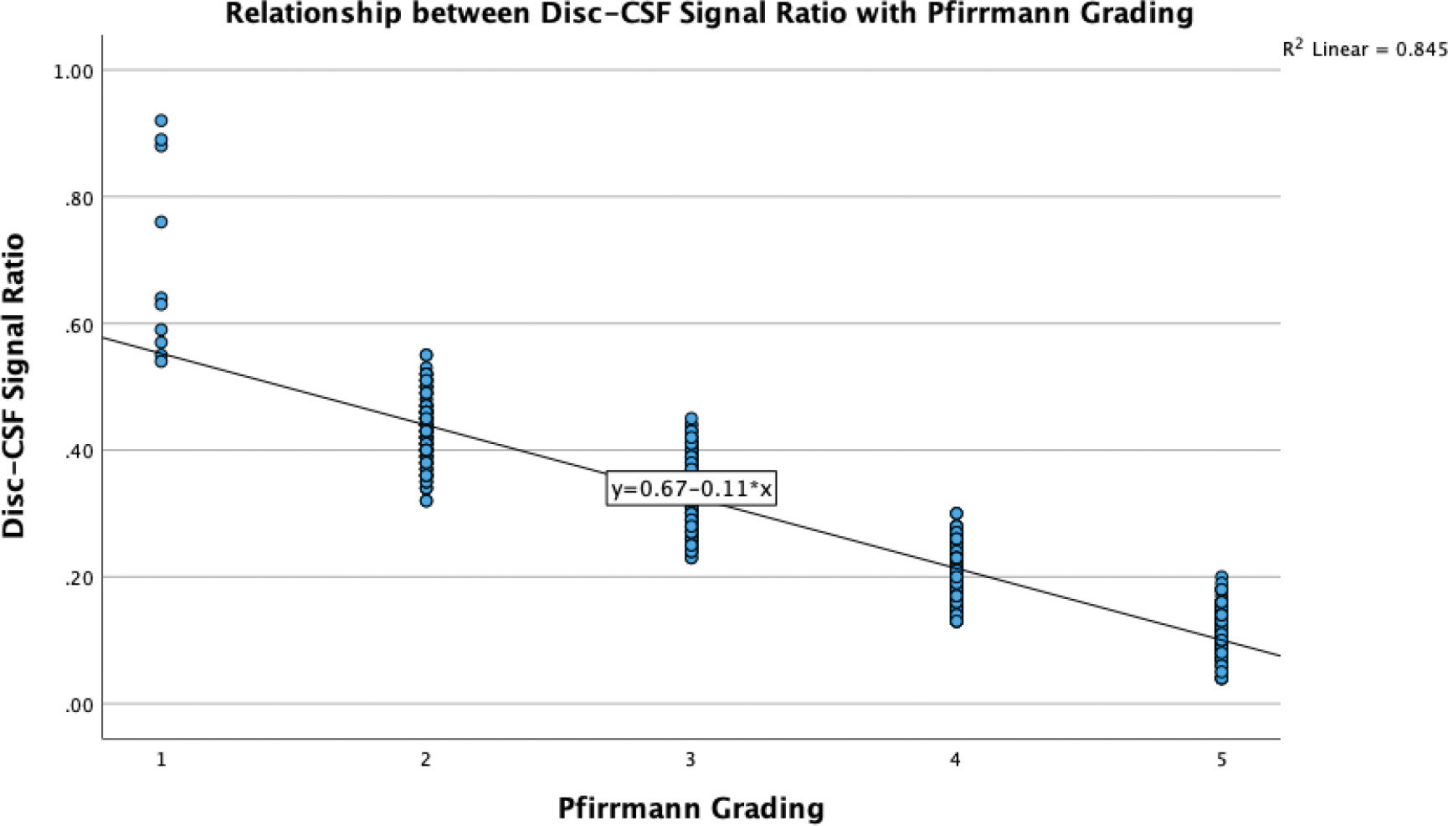
Scatter plot of disc–CSF signal ratio versus Pfirrmann grading.

The progression from grade 1 to grade 5 highlights a clear, inverse relationship between Pfirrmann grades and the DCFR (Table 4). Grade 1 discs, with the highest median DCFR ratio, suggest a well‑hydrated and structurally intact nucleus pulposus. The wide IQR (0.478) may reflect a broader range of early degenerative states within this category. Grade 2 showed a marked decrease in median DCFR, with a very narrow IQR (0.004), indicating consistency in early degenerative changes. Grade 3 continued the downward trend. Whereas, grade 4 and grade 5 exhibited the lowest signal ratios, indicative of severe degeneration with extensive loss of disc material and hydration. This relationship is shown in a scatter plot (Figure 6) to offer a visual confirmation. The plot depicts a clear downward trend, with *R*² value of 0.845 indicating a strong correlation. The data points cluster distinctly for each grade, reinforcing the observation that SI decreases progressively as disc degeneration worsens. The tight clustering of points within each grade further supports the potential use of this DCFR as a diagnostic tool for evaluating disc health in clinical settings.

**Table 4:** Correlation between Pfirrmann grading system and disc–CSF signal ratio.

PFIRRMANN GRADING	DISC–CSF SIGNAL RATIO IN MEDIAN (IQR)
1	0.697 (0.478)
2	0.431 (0.004)
3	0.329 (0.003)
4	0.202 (0.002)
5	0.117 (0.003)

## Discussion

In this study, we investigate lumbar disc degeneration in a Malaysian population using the Pfirrmann grading system alongside the DCFR as a novel measure. Our results demonstrated significant sociodemographic influences, with age and gender prominently affecting disc degeneration severity. As expected, older age groups exhibited more advanced degeneration, while males consistently showed more severe disc degeneration compared to females. Ethnic variations were also observed, with Malay individuals showing 10–15% lower SI compared with their Chinese counterparts. The DCFR provided a reliable, continuous variable for quantifying degeneration, proving especially useful in accounting for differences in MRI imaging conditions.

The effect of gender on the prevalence of disc degeneration has been well documented, with numerous studies highlighting significant differences in how men and women experience this condition. Research suggests that biological, mechanical, and hormonal factors all contribute to these disparities. For instance, studies show that men tend to experience more severe forms of disc degeneration which are in line with our study, possibly due to higher levels of physical activity and occupational loading, which impose greater mechanical stress on the spine [7, 8]. Conversely, some research suggests that women, particularly postmenopausal women, are at greater risk of disc degeneration due to hormonal changes, specifically oestrogen deficiency, which exacerbates the degenerative processes [9]. This hormonal influence might explain why Videman et al. [10] observed a higher incidence of degenerative findings in women that corroborate with the findings in our study.

Quantitative assessments of the mean SI value offer critical insights into the pathogenesis and progression of disc degeneration. Age‑related degeneration is significant, with studies indicating a progressive decline in disc SI and height with advancing age [11]. Our results corroborate with these findings, showing that age significantly determined SI values; with older individuals exhibiting markedly lower median ranks (*P* < 0.001) compared with younger age groups. Additionally, genetic studies have identified the associations between specific gene variants, such as COL9A2, and variations in disc SI, suggesting a genetic predisposition to developing accelerated degeneration [12]. Racial differences were also significant, with Chinese and Indian groups displaying lower median ranks compared with Malays (*P* < 0.001) in our study, potentially due to genetic predispositions [13]. Nevertheless, data on this aspect in the Malaysian context are limited.

Gender‑specific differences in lumbar spine kinematics, as noted by Muriuki et al. [14], can also influence how mechanical loading affects disc health, potentially leading to a variation in the severity of degeneration observed in MRI signal intensities. The study by Dragsbæk et al. [15] highlighted that the association between SI loss and low back pain differs between genders, with women possibly experiencing different clinical manifestations due to factors such as body composition and hormonal differences. Even though the mean SI value of female is lower than male in our study, it does not reach a statistical significance. Overall, while both genders experience disc degeneration, the rate and severity differ, driven by a complex interplay of biological, mechanical, and hormonal factors.

Proteoglycan depletion is a key factor in intervertebral disc degeneration, significantly influencing both hydration levels and MRI signal intensity. Proteoglycans, particularly aggrecan, are crucial for water retention in the nucleus pulposus (NP), ensuring its mechanical integrity. As disc degeneration progresses, a reduction in proteoglycan content diminishes the NP’s capacity to retain water, leading to dehydration and a corresponding decline in MRI signal intensity [16, 17]. Furthermore, the degradation of proteoglycans is exacerbated by inflammatory cytokines like IL‑1β and TNF, which trigger catabolic pathways involving aggrecanases such as ADAMTS‑4, accelerating degeneration [18]. Mechanical stress and oxidative stress also contribute to the breakdown of proteoglycans, further compromising disc integrity [19, 20]. The lower SI of the nucleus pulposus due to decreased proteoglycan content and altered hydration levels, effectively captured by the Pfirrmann grading system [21]. Our findings align with prior research [22, 23] on quantitative MRI assessments of lumbar disc degeneration, particularly the use of SI ratios whereby higher Pfirrmann grades showed significantly reduced signal intensity. Kamei et al. [24] found that the T2 signal ratio was significantly correlated with the Pfirrmann grade and age‑related degeneration, with a 15–20% improvement in consistency over traditional T2WI alone. Contrastingly, studies by Niinimäki [25] highlighted limitations in diffusion‑weighted imaging (DWI), which only showed a 5% difference in apparent diffusion coefficients between degenerated and healthy discs, questioning its clinical utility. Blumenkrantz et al. [26] further demonstrated that T1ρ values could reduce by up to 30% in degenerated discs, suggesting this method’s sensitivity to early biochemical changes. Hence, our DCFR method provides a quantifiable metric and practical alternative without requiring specialized imaging equipment and techniques.

The study acknowledges several limitations that affect the interpretation of the findings. First, the use of a retrospective cross‑sectional design makes it difficult to establish causal links between sociodemographic factors and lumbar disc degeneration. The lack of longitudinal data hinders understanding of the progression of disc degeneration over time. Second, the convenience sampling method potentially introduces selection bias. Although the sample size allowed for statistical analysis, an overrepresentation of the Malay population, limits the generalizability of the findings to other ethnicities and regions. This imbalance limits the statistical power of subgroup analyses particularly the cross‑ethnic comparisons. Future studies with more balanced ethnic representations would be necessary to provide more definitive insights. Despite these limitations, the study contributes important insights into the sociodemographic factors influencing lumbar disc degeneration. This study focuses on a Malaysian cohort, but expanding similar research across different regions could uncover more about the genetic, lifestyle, and environmental factors influencing disc degeneration. Larger multicenter studies with more diverse populations would improve the generalizability of the findings and increase the applicability of the DCFR as a diagnostic tool in wider clinical settings. Longitudinal studies are also needed to track the progression of lumbar disc degeneration over time. It is recommended for future research to explore whether changes in this ratio can predict clinical symptoms of low back pain or the need for surgery, making DCFR useful for both diagnosis and monitoring disease progression.

## Conclusion

This study elucidates the complex interplay between sociodemographic factors and the severity of lumbar disc degeneration. Despite standardized imaging protocols, sociodemographic factors such as age and ethnicity still introduced variability, with older populations showing a 30% greater decline in signal intensity compared with younger cohorts. The Pfirrmann grading system, complemented by signal intensity measurements, provides a robust framework for assessing the severity of lumbar disc degeneration objectively.
